# Back to the Future: Moving Beyond “Mesenchymal Stem Cells”

**DOI:** 10.1002/jcb.23103

**Published:** 2011-03-17

**Authors:** Paolo Bianco

**Affiliations:** Department of Molecular Medicine, Sapienza University of RomeRome, Italy

**Keywords:** Skeletal stem cells, Mesenchymal stem cells, Osteoprogenitors, Pericytes, Bone marrow, Regenerative medicine

## Abstract

The last decade was dominated by dissemination of the notion that postnatal “mesenchymal stem cells,” found primarily in bone marrow but also in other tissues, can generate multiple skeletal and nonskeletal tissues, and thus can be exploited to regenerate a broad range of tissues and organs. The concept of “mesenchymal stem cells” and its applicative implications represent a significant departure from the solidly proven notion that skeletal stem cells are found in the bone marrow (and not in other tissues). Recent data that sharpen our understanding of the identity, nature, origin, and in vivo function of the archetypal “mesenchymal stem cells” (bone marrow skeletal stem cells) point to their microvascular location, mural cell identity, and function as organizers and regulators of the hematopoietic microenvironment/niche. These advances bring back the original concept from which the notion of “mesenchymal stem cells” evolved, and clarify a great deal of experimental data that accumulated in the past decade. As a novel paradigm emerges that accounts for many facets of the biology of skeletal stem cells, a novel paradigm independently emerges for their applicative/translational use. The two paradigms meet each other back in the future. J. Cell. Biochem. 112: 1713–1721, 2011. © 2011 Wiley-Liss, Inc.

The origin of the concept of a “mesenchymal” stem cell goes back to the pioneering experiments of Tavassoli and Crosby in the 1960s [Tavassoli and Crosby, 1968]. While investigating the significance of the specific localization of hematopoiesis in bone, they transplanted boneless fragments of bone marrow into heterotopic sites, and observed the orderly formation of heterotopic bone at the graft site. This revealed that the bone marrow includes an entity, unknown at the time, endowed with the capacity (potential) to generate histology-proven bone tissue. In a series of seminal experiments thereafter, Friedenstein et al. [1974] and Friedenstein [1980] assigned this osteogenic potential first to nonhematopoietic, adherent cells (i.e., to cells corresponding to something within the *stroma* of the bone marrow) and then to cells able to form single cell-derived colonies when grown in culture at low density (i.e., to *clonogenic* stromal cells). The idea that clonogenic stromal cells could be a second class of bone marrow stem cells, distinct from the hematopoietic stem cell, was formulated by Friedenstein [1990] and Owen and Friedenstein [1988] based on the observation that heterotopic transplants of cell strains originating from a single clonogenic cell could generate a variety of tissues; that is, bone-forming osteoblasts, cartilage-forming chondrocytes, adipocytes, and fibroblasts. These experiments proved multipotency of single clonogenic bone marrow stromal cells, and their ability to generate differentiated phenotypes, each of which corresponded to one elemental histological feature of a skeletal segment. This idea rested on solid experimental evidence, which in turn was centered on the use of in vivo transplantation assays as the way to assess differentiation potential. Tissues formed under defined experimental circumstances were rigorously histology-proven, leaving no ambiguity as to the genuine capacity of grafted cells to generate differentiated tissues. There was no need to expose cells to differentiating cues ex vivo in order to prove or probe their differentiation potential.

The idea of a stem cell for connective tissues was indeed quite revolutionary. The idea that such stem cell would be found in the bone marrow added extra charm, given the known identity of the bone marrow as the site where the best-known stem cell, the hematopoietic stem cell, is found. The idea remained known, however, only to experimental hematologists and skeletal biologists, for quite a long time. However, the idea had precise boundaries: the putative stem cell was a common progenitor of skeletal tissues, not of all mesoderm derivatives; and it was found in the bone marrow, not everywhere. The idea of a “mesenchymal” stem cell [Caplan, 1991; Pittenger et al., 1999] was directly based on the body of knowledge generated by the work of Friedenstein et al.; however, it was a different idea. This idea reads that the putative “mesenchymal” stem cells is a common progenitors, not just of skeletal tissues, but of “mesenchymal” tissues, meaning virtually all nonhematopoietic derivatives of mesoderm; and although found in the bone marrow, it is not unique to the bone marrow.

Facilitated by the concurrent explosion of interest in stem cells at large, in turn potently fueled by the isolation of human embryonic pluripotent cells in culture, the idea of a “mesenchymal stem cell” in postnatal tissues gained fast, widespread acceptance. However, it remained essentially unproven. In addition, certain implications of the idea that blatantly collide with known facts of developmental biology were pushed in the back, and several thousands of papers published in the last decade all unitedly claim as an established fact that, for example, “mesenchymal stem cells” give rise to skeletal muscle *and* bone. Myogenic potential, instead, is highly restricted to somites, whereas a skeletogenic potential is found in axial and lateral mesoderm, alike, which give rise to axial and limb bones, respectively, and even in ectoderm (neural crest)-derived cells that give rise to the craniofacial bones. After spatial specification of mesoderm, there is no “common progenitor” even for bone cells of different skeletal segments, and no “mesenchymal stem cell” that is, *both* myogenic and skeletogenic in the embryo. Why and wherefrom should there be such a common progenitor in postnatal tissues is not easily explained by developmental biology.

Two specific facts contributed significantly to generate the widespread, as much as nebulous notion, that there are progenitors of virtually all mesoderm derivatives, virtually everywhere in the postnatal organism. One is the kind of assays used. In vitro assays based on exposure of cultured cells to artificial “differentiative” cues, followed by assessment of a handful of markers, do not have the same stringency as demonstrating generation of histology-proven tissues with no ex vivo cueing of differentiation. The other is the use, most often inadvertent, of factors that do in fact reprogram a cell's fate, the most commonly used being BMPs. Spontaneous differentiation potential, and responsiveness to reprogramming, are equally important biological characteristic of a given cell, and yet they are radically distinct conceptually, and experimentally. Thus, the ability of a myogenic cell to generate osteoblasts upon treatment with BMPs is significant, but does not signify a differentiation potential of the same kind as that of a cell that can generate osteoblasts and bone with no need of BMPs. It is, essentially for these reasons, that the conservative use of the term *skeletal stem cells* was recommended to refer to bone marrow-derived, multipotent stromal cells with an in vivo assayable osteogenic potential [Bianco et al., 2006, 2008].

## THE TWO UNSOLVED QUESTIONS

Friedenstein's work left two key questions unaddressed: one was the in situ counterpart of the explanted, clonogenic, and multipotent stromal cells regarded as a putative stem cell. The other was the evidence that the multipotent cells could also self-renew, and therefore be truly regarded as bona fide stem cells. The first question had remained unaddressed essentially due to the lack of markers suited to bridge the gap between observations made ex vivo and in vivo (both in the intact bone marrow and in tissues formed by transplantation). Most markers identified over time since the pioneering generation of the Stro-1 antibody [Simmons and Torok-Storb, 1991] were essentially employed to enrich prospectively the subset of stromal cells endowed with clonogenicity, but not for identifying where cells that could be explanted originate from bone marrow. The second question was confounded, again, by the universal use of in vitro—only experimental approaches. Self-renewal cannot be reliably proven in vitro (reviewed in Bianco et al. [2008]), and the concept of self-renewal became widely confused with the mere ability of a given cell to initiate long-term, extensive proliferation in culture. The number of population doublings in culture became, in the mind of many, a token of self-renewal capacity, in spite of the known fact that the only kind of postnatal stem cell for which self-renewal was ever conclusively proven (hematopoietic stem cells) do not expand, proliferate or double ex vivo at all, and their self-renewal was proven in vivo and in vivo only. So much so, that the dependence of HSCs from the in vivo environment for their self-renewal became incorporated into another totemic notion of stem cell biology, the concept of a “niche” [Schofield, 1978].

## IDENTITY OF SKELETAL STEM CELLS

Skeletal stem cells appear to coincide with a cell type long known in classical histology, but largely left neglected due to the inherent difficulty in visualizing it under standard microscopy. Adventitial reticular cells [Weiss, 1976; Westen and Bainton, 1979] are slender, elongated cells residing over the abluminal surface of sinusoids, and regularly missed in standard histological images. They can be demonstrated in situ by ALP reactivity provided that the sample has been processed in specific ways [Westen and Bainton, 1979; Bianco et al., 1988; Bianco and Boyde, 1993], or by immunoreactivity for MCAM/CD146 [Sacchetti et al., 2007]. MCAM is a cell adhesion molecule of the immunoglobulin superfamily, also expressed in subsets of endothelial cells, and in a restricted range of other cell types [Shih, 1999]. It is regulated by Notch signaling, and mediates interactions with an unknown ligand. If used in cell sorting experiments of freshly isolated cells, MCAM surface expression identifies, like other surface epitopes (e.g., Stro-1) all clonogenic stromal cells [Sacchetti et al., 2007]. Its expression is retained in culture, and lost upon osteoblastic differentiation; following transplantation, only stromal cells that re-establish a close anatomical association with local, nascent blood vessels express MCAM [Sacchetti et al., 2007]. Ultimately, these cells reform cells with the typical morphology, position, and association with individual hematopoietic cells that defines adventitial reticular cells. If extracted from heterotopic ossicles, these cells behave like CFU-Fs [Sacchetti et al., 2007].

In vitro, bone marrow stromal cells (BMSCs, CD146^+^ adventitial reticular cells) exhibit a unique phenotype. Global analysis of their transcriptome reveals the co-expression of sets of genes that characterize early osteogenic progenitors (but not mature osteoblasts) on the one hand, and mural cells/pericytes on the other [Sacchetti et al., 2007]. In addition, cultured BMSCs respond to known regulators of mural cell proliferation or quiescence [Hirschi and D'Amore, 1996, 1997] in a way consistent with their mural cell nature; that is, they are induced to proliferate by FGF-2, they are induced to quiescence by TGF-beta; they robustly express endothelial differentiation gene (EDG) receptors, which are necessary for pericyte recruitment, and regulate their expression in response to pericyte mitogens or anti-mitogens [Sacchetti et al., 2007]. Both in vitro and in vivo, BMSCs are potent producers of angiopoietin-1, which is a known product of pericytes [Suri et al., 1996], a known regulator of primary microvascular remodeling [Suri et al., 1996], and also a regulator of HSC quiescence in their bone marrow niche [Arai et al., 2004]. In keeping with a role in HSC regulation, BMSCs also express virtually all genes that have been implicated in regulation of HSCs, such as N-cadherin and Jagged-1 [Sacchetti et al., 2007]. In keeping with their ability to establish a hematopoietic microenvironment, and to interact with HSCs, BMSCs (“mesenchymal stem cells”) are increasingly seen as directly implicated in regulation of HSCs, or else, in providing a “niche” for them [Sacchetti et al., 2007; Mendez-Ferrer et al., 2010; Omatsu et al., 2010; Raaijmakers et al., 2010]. Importantly, properties recognized for human bone marrow skeletal stem cells (i.e., the archetypal “mesenchymal stem cells” in humans) are duplicated in murine BM “MSCs,” in turn identified as perivascular, self-renewing osteoprogenitors [Mendez-Ferrer et al., 2010].

## SELF-RENEWAL OF SKELETAL STEM CELLS

Self-renewal is the ability of stem cells to maintain the stem cell pool while generating progenies that undergo clonal expansion and differentiation (reviewed in Bianco et al. [2008]). Admittedly linked to environmental cues found in defined tissue “niches,” self-renewal implies a kinetically (possibly also physically) asymmetrical proliferation [Watt and Hogan, 2000; Bianco et al., 2008]. Demonstrating self-renewal implies that a minimum phenotype of the candidate stem cell is defined, as this must be recognized in order to recognize the self-renewing stem cell itself; it also implies an in vivo assay that directly demonstrates self-renewal in the context of tissue reconstitution. As widely known, proof of self-renewal for HSCs is given by the ability of prospectively isolated, phenotype-defined cells to reconstitute hematopoiesis under limiting conditions in vivo, serially [Bianco et al., 2008]. Skeletal stem cells can self-renew inasmuch as they can be explanted as MCAM-expressing adventitial reticular cells/CFU-Fs, grown through several population doublings, and then transplanted to reconstitute a compartment of identical cells in vivo while generating heterotopic ossicles [Sacchetti et al., 2007]. We now know that human “MSCs” can at least be serially passaged [Sacchetti et al., 2007], and murine “MSCs” can be serially transplanted [Mendez-Ferrer et al., 2010]. For these reasons, whether called “skeletal” or “mesenchymal” depending on the measure of rigor versus compliance with the popular notion one wants to meet, nonhematopoietic stromal skeletal progenitors found in human and murine bone marrow are bona fide stem cells. What should not be missed, however, is the physical dimension of their self-renewal revealed by heterotopic transplantation assays: this coincides with the physical association of MCAM-expressing cells with the wall of nascent sinusoids. It is through the interaction with endothelial cells that mesenchymal cells recruited to a mural cell fate become quiescent [Antonelli-Orlidge et al., 1989; Hirschi and D'Amore, 1996; Jain, 2003], and quiescence within a “niche” is a defining feature of stem cells.

## POTENCY OF SKELETAL STEM CELLS

Three important tenets of experiments designed to probe the inherent differentiation potential of skeletal stem cells are commonly overlooked, and therefore never reiterated enough. One is that any differentiation assay used to claim multipotency must be conducted with clonal populations of cells. The second is that any single differentiation potency must be probed under conditions that exclude known or potential reprogramming effects. The third is that differentiation must be unequivocal; that is, not merely based on expression of a handful of tissue-characteristic proteins or mRNAs (or even artifactual events such as dystrophic mineral deposition at largely unphysiological phosphate concentrations), and ideally coinciding with generation of histology proven tissue in vivo. In a nutshell, multipotency must be probed *clonally*, and any claimed potency must be *native* and *robust*. When actual data, and common wisdom on the differentiation potency of “mesenchymal” stem cells is gauged with these criteria, a number of commonplace assumptions fade away. Ability of “MSCs” to form bone in vivo, with no induction, is not universally found in different tissues. Ability of to form cartilage in vivo (and even in vitro) is not universally found even in bone marrow-derived “MSCs,” regardless of their in vitro and in vivo history (e.g., donor age, passage number), and independent of other determinants (such as oxygen tension and cell density, commonly assumed to play a key role in dictating outcome of individual assays). Most importantly, the ability to turn on one or more “osteoblastic” or “adipogenic” traits in vitro does not predict true bone or fat formation in vivo [Satomura et al., 2000; Bianco et al., 2001; Sacchetti et al., 2007].

## THE MICROVASCULAR NATURE OF SKELETAL STEM CELLS

A close link between the microvasculature and tissue progenitors had been surmised from multiple, independent lines of evidence. Diaz-Flores et al. 1990, 1991a, b, 1992 had showed that microvessels of skeletal and periskeletal tissues were associated with osteo-chondroprogenitors, tentatively identified as pericytes; Canfield and coworkers had also provided evidence that vascular pericytes were endowed with some osteogenic potential [Doherty et al., 1998; Doherty and Canfield, 1999]; and it had been noted that bone marrow stromal cells in situ are in fact perivascular cells, coinciding with adventitial reticular cells [Bianco and Boyde, 1993; Bianco and Gehron Robey, 2000; Bianco et al., 2001]. Recognition of bone marrow clonogenic progenitors of skeletal tissues are indeed self-renewing, bona fide stem cells, and are indeed associated with marrow sinusoids thus closed the circle, and addressed at one time the two questions left open at the end of Friedenstein's work. In the view of those who had endorsed the “mesenchymal stem cell” paradigm, this then raised the question—are “*all* mesenchymal stem cells pericytes?” [Caplan, 2008]. Thus, the identical approach used to prospectively isolate the archetypal “mesenchymal stem cells” from bone marrow was borrowed to isolate “mesenchymal stem cells” from other tissues [Crisan et al., 2008], which led to suggest that yes, all “mesenchymal stem cells” from all tissues, are all pericytes, and they all form virtually all mesoderm derivatives. While this view collides with other published reports, the question remains entirely open to direct experimentation whether “pericytes” (microvascular nonendothelial cells) from different tissues are equipotent or not. Extreme rigor and accurate choice of assays in tackling this question is of paramount importance.

**Fig. 1:**
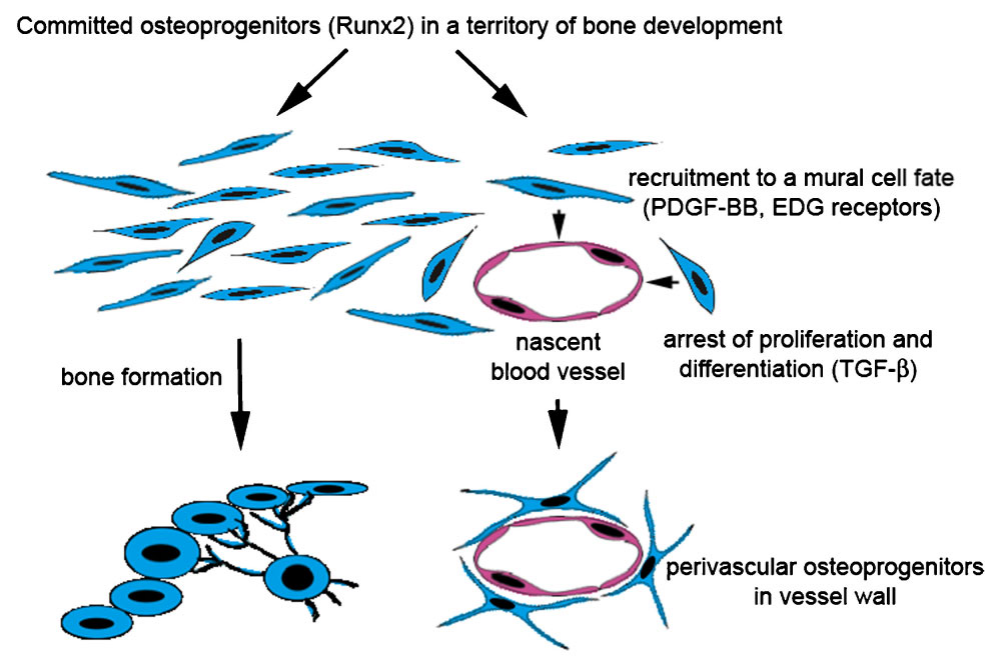
Diagram illustrating a model of the origin of postnatal skeletal progenitors. As blood vessels develop and grow within a field of bone organogenesis, committed (Runx2 expressing) osteoprogenitors interact with the endothelial cells of the vessel wall. PDGF-BB produced by endothelial cells signals through PDGF-Rβ expressed in mesenchymal cells. Mesenchymal cells (which express VEGF and Ang-1, thereby influencing growth and remodeling of nascent vascular lattices) are recruited to the vessel wall, where they are induced to mitotic quiescence and arrest of differentiation. They become mural cells (adventitial cells), with a native osteogenic potential, and a residual potential for further growth and differentiation. In this model, similar events might mediate the recruitment of other tissue-specific, committed progenitors to vascular walls in other tissues (e.g., committed myogenic progenitors could be recruited to microvascular walls in developing skeletal muscle). Interaction of presumptive mural cells with vessel walls might involve MCAM/CD146, and adhesion molecule expressed in skeletal stem cells and in mural cells/pericytes of other tissues as well.

The notion that skeletal (“mesenchymal”) progenitors are located perivascularly, in and of itself, provides not just a clue to anatomy, but, for the first time, a clue to developmental origin of the cells in question. With respect to bone marrow “mesenchymal stem cells,” it has been claimed previously that they are in fact *committed* skeletogenic progenitors [Bianco et al., 2008], as suggested not only by their native skeletogenic potential as probed in vivo, but by the constitutive expression of the master regulator of skeletogenesis, Runx2 [Satomura et al., 2000; Sacchetti et al., 2007]. Combining these notions with simple appraisal of how the bone marrow develops, it is easy to recognize that osteogenic cells pre-exist, in development, the appearance of bone marrow cavity, of bone marrow hematopoiesis, bone marrow stroma, and bone marrow stromal cells [Bianco et al., 1999]. Thus, it was previously argued that a close association of primitive osteogenic cells (found in the perichondrium) with blood vessels invading the nascent marrow cavity would be the event leading to the establishment of skeletogenic stromal cells in the marrow cavity [Bianco et al., 1993, 1999], a view strongly supported by recent stringent evidence [Maes et al., 2010]. This view is fully consistent with what is known of the origin of subendothelial mural cells in general, and in all tissues from local mesenchymal cells residing in the vicinities of developing blood vessels [Hirschi and D'Amore, 1996; Jain, 2003], Figure 1. While predicting that bone marrow mural cells would be osteogenic if properly assayed, this also predicts that natively osteogenic cells would be found as mural cells in bone (marrow), but in bone only. On the other hand, the general nature of regulated mural cell recruitment as a developmental event in all tissues, would predict that tissue-specific, committed progenitors found in nonskeletal tissues would be also recruitable to a mural cell fate [Bianco et al., 2008]. Thus, one would, for example, find committed myogenic cells in microvascular walls of skeletal muscle, not endowed with a native osteogenic potential unless exposed to BMPs. This, again, has been observed. Human muscle includes a class of myogenic pericytes, which can express at least some osteogenic features, but only upon exposure to BMPs [Dellavalle et al., 2007]. However, when transplanted in vivo using assays geared to gauge the native osteogenic potential of bone marrow skeletal progenitors, these cells do not form bone. From these data, and pending more extended analysis, the hypothesis was formulated that microvascular walls would include, in each tissue, a defined population of tissue-specific committed progenitors [Bianco et al., 2008]. This system would be made of cells with a similar and yet nonidentical phenotype, in which markers of mural cells would be shared across tissues. This view would replace the paradigm of “mesenchymal stem cells” as a uniform population of ubiquitously distributed, multipotent, equipotent cells with broad potential [Crisan et al., 2008], and would for the first time be rooted into a recognizable developmental pathway. This pathway would account for the very existence of postnatal progenitors of mesoderm-derived tissues other than blood. In this view, blood vessels would act, during development and growth, as “traps” of tissue progenitors. Some of these would be randomly recruited to a mural cell fate due to their proximity to ingrowing blood vessels, and thereby be diverted from completion of their proliferative and differentiative fate, and rather, be retained as quiescent, committed but not differentiated cells. This process could be operating at multiple times and developmental ages. In prenatal development, it is known for example, that somite-derived mesenchymal sharing a clonal origin with myogenic cells join the wall of the dorsal aorta, with which they physically associate prior to differentiating into smooth muscle [Esner et al., 2006]. This fact might be related to the existence of myogenic [De Angelis et al., 1999] and skeletogenic [Minasi et al., 2002] progenitors in the wall of the embryonic aorta, which led to formulate the hypothesis of “mesoangioblasts” [Minasi et al., 2002]. Interestingly, in experiments in which quail “mesoangioblasts” were transplanted to chick developing wings, grafted cells did contribute to a variety of tissues, but by far the most robust contribution was to the adventitia of local, growing blood vessels [Minasi et al., 2002]. At hatching, quail cells were found within a variety of tissues, but the largest numbers of adventitial quail cells were found over vascular branches of every order from large arteries down to pericyte-coated precapillary arterioles [Minasi et al., 2002]. In search for a potential postnatal correlate of mesoderm progenitors apparently associated with the embryonic dorsal aorta, one initial attempt identified alkaline phosphatase as a candidate marker suited to isolate myogenic progenitors from human skeletal muscle microvessels [Dellavalle et al., 2007]; it was later shown that MCAM/CD146, which identifies bone marrow skeletal progenitors [Sacchetti et al., 2007], also identifies muscle microvascular myogenic progenitors [Crisan et al., 2008]; however, whether the ALP^+^ population and the CD146^+^ population of myogenic cells are equivalent to one another, or to bone marrow skeletal progenitors that express both markers, is far from being conclusively settled. More in general, the view that pericytes found in nonmuscle tissue are uniformly myogenic and osteogenic, and thus represent the in situ correlate of a ubiquitous and equipotent “mesechymal stem cell” [Crisan et al., 2008], needs to be carefully scrutinized.

## DETERMINED AND INDUCIBLE MICROVASCULAR PROGENITORS

The general idea underlying the use of the term “mesoangioblast” (tailored on the term hemoangioblast, already in existence to denote a common progenitor of endothelial and hematopoietic cells [Cossu and Bianco, 2003]), actually implied that conversion of endothelial cells, or of endothelial progenitors, to a mesenchymal progenitors could occur [Bianco and Cossu, 1999], and contribute to an unknown extent to generate extravascular mesoderm derivatives [Bianco and Cossu, 1999; Cossu and Bianco, 2003]. While the significance of these putative events in physiology remains uncertain, strong support of the notion that these events can significantly operate in disease came recently, with the demonstration that TGF-beta2 and BMP4 can direct the conversion of endothelial cells to osteochondrogenic cells, and that this is mediated by ALK2 [Medici and Olsen, 2011; Medici et al., 2010]. As ALK2 mutations underlie human fibrodysplasia ossificans progressiva (FOP), a devastating disorder in which muscles turn into bone, a source of the osteochodrogenic cells that abnormally differentiate within skeletal muscle in FOP is thereby identified. In the light of this, FOP becomes a disease of endothelial cells [Medici et al., 2010], in which abnormal BMP signaling mediated by ALK2 promotes endothelial to mesenchymal conversion. The classical notion that BMP can induce heterotopic bone formation in muscle finds in these novel data a cellular target and effector. The equally classical notion of two classes of skeletogenic progenitors (determined and inducible) becomes clarified. Friedenstein's “determined” and “inducible” progenitors [Friedenstein, 1968] would coincide with cells not requiring, or vice versa requiring, BMP-mediated reprogramming to a skeletogenic potential, and endothelial cells would become a prime, but likely not the sole, member of the “inducible” skeletogenic cells. Interestingly, both classes of progenitors would be integral to microvascular walls, at least in muscle.

## THE ANGIOPOIETIC FUNCTION OF “MSCs”

Analysis of the developmental sequence whereby heterotopic hematopoiesis is established after transplantation of human skeletal stem cells led to the recognition of their role in guiding the development of a local system of sinusoids, replicating the sinusoidal type of microvasculature characteristic of the bone marrow [Sacchetti et al., 2007]. In these experiments, formation of sinusoids clearly follows the establishment of bone and precedes the establishment of hematopoiesis, replicating the discrete steps observed in the natural development of a bone rudiment. A close interaction of skeletal stem cells and endothelial cells can be duplicated by in vitro assays [Sacchetti et al., 2007], in which formation of pseudovascular cords is directed by skeletal stem cells, and by in vivo assays, in which endothelial cells are co-transplanted with “mesenchymal” stem cells. Initially performed with C3H10T1/2 cells [Koike et al., 2004], which are not a direct equivalent of postnatal bone marrow “mesenchymal” stem cells, these in vivo experiments showed that endothelial cells and “MSCs” could assemble a fully functional network of capillaries in collagenous carriers. Similar results were obtained in later experiments in which bone marrow “MSCs” were substituted for murine embryonic cells [Au et al., 2008; Melero-Martin et al., 2008]. This phenomenon portrays a hitherto overlooked function of “MSCs”—their ability to guide the assembly of functional blood vessels given an efficient local number of endothelial cells. This phenomenon is quite distinct from the two canonical developmental events that lead to the appearance of new blood vessels. It is distinct from angiogenesis, as it not dependent on growth of pre-existing vessels, and it is distinct from vasculogenesis as it does not depend on de novo differentiation of endothelial cells. “Mesenchymal” stem cells can direct the formation of blood vessels given a sufficient supply of differentiated endothelial cells. We like to refer to this property as “angiopoiesis,” to denote its distinction from both angiogenesis and vasculogenesis. An angiopoietic function of “MSCs” is consistent with their nature as “mural cells”/pericytes, with the expression of genes mediating pericyte characteristic functions such as angiopoietin-1 and many more, and with the manner in which they respond to factors mediating endothelial–mural cells interactions. Important question remain to be addressed in this connection. One is whether the same function is shared by “MSCs” from bone marrow and nonbone marrow tissues, and to what extent; another is the role of MCAM/CD146, which is apparently shared in mural cells in microvascular districts of multiple tissues [Crisan et al., 2008], in this function. As a cell adhesion molecule, MCAM/CD146 is a natural candidate in mediating interactions of “mesenchymal stem cells” with other cells. As the closest neighbor of “MSCs” in situ, endothelial cells are natural candidate partners in these interactions. Some evidence for the perturbation of this interaction using knockdown of MCAM/CD146 has been provided in vitro [Sacchetti et al., 2007], and similar evidence is in the process of being pursued by in vivo assays. Finally, one intriguing implication of available data is that bone marrow “MSCs” appear to guide the formation of sinusoids, rather than capillaries, under conditions in which their osteogenic potential can unfold (as permitted by the use of mineral-based, hard, osteoconductive scaffolds); they guide the formation of capillaries, rather than sinusoids, unlike when soft scaffolds are used, and bone formation is barred. Careful analysis of this divergent behavior might shed light into the mechanisms dictating the formation of a sinusoidal-type microvascular network, a key developmental event in hematopoiesis. Overall, the search for the cellular identity of skeletogenic progenitors under normal and pathological conditions is revealing an intricate scenario, and is unquestionably placing blood vessels at center stage.

## SKELETAL (MESENCHYMAL) STEM CELLS AND REGENERATIVE MEDICINE

Novel views of the biological functions of skeletal stem cells intertwine with emerging paradigms for clinical translation of the properties of “mesenchymal” stem cells. The past decade was dominated by the hypothesis that “mesenchymal” stem cells could generate not only all mesoderm-derivatives, including skeletal muscle, heart, and endothelial cells, but perhaps even derivatives of other germ layers, such as neurons or liver cells (reviewed in Bianco et al. [2008, 2010]). These suggestions were heavily influenced by a climate in which the isolation of human embryonic pluripotent cells in culture had fueled hopes and controversies. “Stem cells” had become in the mind of many investigators, as much as in the lay view, a uniform entity with “embryonic” and “adult” subsets. As a result, the dominant view of the significance of stem cells for medicine was that of tissue and organ regeneration and substitution, to be accomplished through the virtues of pluripotent or broadly multipotent cells, of which “mesenchymal stem cells” would represent a prime subset. Suggestions of a strikingly broad differentiation potential of “MSCs,” which would make them a substitute of embryonic pluripotent cells for mechanically regenerating a number of unrelated tissues, have not held to their promises. While the claimed ability of “MSCs” (or of any other kind of tissue-specific stem cells including hematopoietic stem cells) to regenerate heart muscle, for example, has not been confirmed, we have witnessed transplanted bone marrow-derived “MSCs” make bone within the heart [Breitbach et al., 2007], in keeping with their natural, true potential, and known in vivo performance. Meanwhile, a quite substantial body of experimental data have indicated some beneficial effect exerted by “MSCs” on the repair of nonskeletal tissues. As noted [Prockop, 2007], these effects cannot be accounted for, or even be seen as dependent on, the differentiation potential of “MSCs,” and must rely on other properties thereof. In this context, the angiopoietic function of “MSCs” might deserve some attention. A contribution to the organization of a local network of newly formed capillaries may well underlie beneficial effects seen in experiments in which “MSCs” are used for repairing organs and tissues that are not germane to their lineage and differentiative potential (such as heart and brain). Along this line, some of the “trophic” effects evoked for “MSCs” [Caplan and Dennis, 2006] employed in translational studies could be traced back to the very function that bone marrow “MSCs” exert physiologically. Likewise, the immunomodulatory effects of “MSCs” reflected in their use for treating acute graft vs. host disease [Le Blanc and Ringden, 2007], which are quite departed conceptually from their originally envisioned use for bone regeneration, are not departed from the hematopoietic regulatory function of “MSCs.” As providers of the “hematopoietic” microenvironment, bone marrow stromal cells serve a highly differentiated function centered on regulation of another cell's growth and differentiation—a “trophic” effect indeed. Therefore, a new way of seeing the potential use of “MSCs” for treating losses of nonskeletal tissues might for the first time be placed on a basis more rational than the never conclusively proven ability of “MSCs” to generate nonskeletal tissues. In this respect, studies elucidating the nature and function of “MSCs” as tissue organizers are by default elucidating how to proceed with their use for clinical translation.

Inscribed in this context is the emerging role of “MSCs” as stem cell niches (reviewed in Bianco, in press). The ability of “MSCs” to act as dynamic organizers of the hematopoietic microenvironment [Sacchetti et al., 2007] paved the way for a number of studies that redirected attention from osteoblasts and endothelial cells of sinusoids (first cell types to be implicated as “niche” cells in bone marrow, reviewed in Bianco, in press) to “MSCs” or osteoprogenitors as providers of the “niche” effect for hematopoietic stem cells [Chan et al., 2009; Mendez-Ferrer et al., 2010; Omatsu et al., 2010; Raaijmakers et al., 2010]. As a stem cell directing the behavior of another stem cell, skeletal stem cells come into light as a prime example of a hitherto overlooked biological phenomenon—the interplay of two distinct kinds of stem cells at a single spatial tissue specification [Bianco, in press; Sacchetti et al., 2007]. This notion, appealing per se for its biological significance, again conveys a novel angle on applicative, translational approaches involving the use of, or the focus on, skeletal stem cells. Attempts have already been made to manipulate the HSC “niche” using regulators of the physiology of skeletal lineage such as PTH [Calvi et al., 2003], in order to optimize physiological interactions leading to homing and engraftment of transplanted HSCs. Genetic manipulation of skeletal progenitors can disrupt the conservative kinetics of HSC self-renewal, leading to myelodysplasia and leukemogenesis [Raaijmakers et al., 2010]. Control of hematopoietic physiology by skeletal stem cells thus opens highly innovative prospects for understanding and targeting hematopoietic disease [Adams and Scadden, 2008; Lane et al., 2009]. Likewise, the hematopoietic microenvironment per se (the “soil” in a commonplace paradigm) is hijacked by blood-borne hematopoietic and nonhematopoieitc cancer cells (the “seed” in the same paradigm) such as in leukemia or skeletal metastasis. Curiously, “mesenchymal stem cells” have received attention as related to cancer biology and the metastatic process specifically, mostly as acting within a primary, extraskeletal cancer and promoting its invasive/metastatic behavior [Karnoub et al., 2007]. The role of skeletal progenitors in facilitating homing and “engraftment” of cancer cells to the bone environment has received comparatively less attention. However, the process of establishing cancer growth in the bone/bone marrow environment shares fundamental dynamics with the process of establishing hematopoiesis in bone, in which skeletal stem cells clearly play a major role. Elucidating the role of skeletal progenitors in providing the cancer microenvironment in bone will thus open new ways of conceiving intervention in skeletal metastasis, currently centered on the interplay of cancer with differentiated bone cells such as osteoblasts and osteoclasts, rather than with the bone marrow stroma proper.

As the general view of the relevance of skeletal progenitors for medicine at large is shifting, so is the view of their use for skeletal diseases proper. Originally viewed as the fundamental bricks for reconstructing bone in a fundamentally surgical scenario, skeletal progenitors are at the same time the mediators of bone disease as diseases of the osteogenic lineage [Bianco and Robey, 1999]. Their kinetics, regulation, and function stands at the core of all skeletal diseases involving a dysfunction of bone-forming cells proper. Indeed, the view of skeletal stem cells as players and models of bone disease had been proposed long before the modeling of disease through pluripotent stem cells gained broad attention. The ability of transplanted, mutated skeletal stem cells to generate miniature replicas of human abnormal bone [Bianco et al., 1998] and the analysis of the natural history of certain genetic diseases as rooted into the kinetics of the skeletal lineage and its stem cells [Kuznetsov et al., 2008] provide a relevant example. More advances can be expected to come from viewing stem cells not as tools, but as targets of therapy.

Beyond bone reconstruction, the very notion of skeletal stem cells evokes the ability to tackle diseases that have no cure, and among these, genetic disease of the skeleton. Initial approaches involved transplantation procedures directly borrowed from modes and concepts specific to hematopoietic stem cell grafting, but not germane to the nature and properties of a different lineage, a different system, and a different stem cell. This was the case in attempts to correct osteogenesis imperfecta by bone marrow transplantation [Horwitz et al., 2001]. As technologies make it possible to correct genetic defects within skeletal stem cells, modes of intervention based on gene therapy in stem cells become at least conceivable [Bianco et al., 2010]. Refined and safer ways of approaching gene therapy at large, circumventing some of the present-day hurdles, will need to be met in the future, by a proper know-how on handling these approaches for skeletal stem cells specifically. Evidence that at least some of the adverse effects of a human disease-causing mutation can be reversed in skeletal stem cells in vitro is but the first step in this direction [Piersanti et al., 2010].
